# Berberine may rescue *Fusobacterium nucleatum*-induced colorectal tumorigenesis by modulating the tumor microenvironment

**DOI:** 10.18632/oncotarget.5166

**Published:** 2015-09-12

**Authors:** Ya-Nan Yu, Ta-Chung Yu, Hui-Jun Zhao, Tian-Tian Sun, Hui-Min Chen, Hao-Yan Chen, Hui-Fang An, Yu-Rong Weng, Jun Yu, Min Li, Wen-Xin Qin, Xiong Ma, Nan Shen, Jie Hong, Jing-Yuan Fang

**Affiliations:** ^1^ State Key Laboratory of Oncogenes and Related Genes, Division of Gastroenterology and Hepatology, Renji Hospital, School of Medicine, Shanghai Jiaotong University, Shanghai Cancer Institute, Shanghai Institute of Digestive Disease, Shanghai, China; ^2^ Department of Gastroenterology, the Affiliated Hospital of Qingdao University, Qingdao, Shandong Province, China; ^3^ Shanghai Majorbio Bio-pharm Biotechnology Co. Ltd., Shanghai, China; ^4^ Department of Medicine & Therapeutics, State Key Laboratory of Digestive Disease, Institute of Digestive Disease and LKS Institute of Health Sciences, CUHK Shenzhen Research Institute, The Chinese University of Hong Kong, Shatin, Hong Kong; ^5^ Department of Clinical Laboratory, Renji Hospital, School of Medicine, Shanghai Jiaotong University, Shanghai, China; ^6^ State Key Laboratory of Oncogenes and Related Genes, Shanghai Cancer Institute, Renji Hospital, Shanghai Jiao-Tong University School of Medicine, Shanghai, China; ^7^ Department of Rheumatology, Renji Hospital, School of Medicine, Shanghai Jiaotong University, Shanghai, China

**Keywords:** colorectal tumorigenesis, intestinal microbiota, fusobacterium nucleatum, berberine, tumor-immune cytokine

## Abstract

**Background:**

Accumulating evidence links colorectal cancer (CRC) with the intestinal microbiota. However, the disturbance of intestinal microbiota and the role of *Fusobacterium nucleatum* during the colorectal adenoma-carcinoma sequence have not yet been evaluated.

**Methods:**

454 FLX pyrosequencing was used to evaluate the disturbance of intestinal microbiota during the adenoma-carcinoma sequence pathway of CRC. Intestinal microbiota and mucosa tumor-immune cytokines were detected in mice after introducing 1,2-dimethylhydrazine (DMH), *F. nucleatum* or Berberine (BBR), using pyrosequencing and Bio-Plex Pro™ cytokine assays, respectively. Protein expressions were detected by western blotting.

**Results:**

The levels of opportunistic pathogens, such as *Fusobacterium*, *Streptococcus* and *Enterococcus* spp. gradually increased during the colorectal adenoma-carcinoma sequence in human fecal and mucosal samples. *F. nucleatum* treatment significantly altered lumen microbial structures, with increased *Tenericutes* and *Verrucomicrobia* (opportunistic pathogens) (*P* < 0.05 = in wild-type C57BL/6 and mice with DMH treatment). BBR intervention reversed the *F. nucleatum*-mediated increase in opportunistic pathogens, and the secretion of IL-21/22/31, CD40L and the expression of *p*-STAT3, *p*-STAT5 and *p*-ERK1/2 in mice, compared with mice fed with *F. nucleatum* alone.

**Conclusions:**

*F. nucleatum* colonization in the intestine may prompt colorectal tumorigenesis. BBR could rescue *F. nucleatum*-induced colorectal tumorigenesis by modulating the tumor microenvironment and blocking the activation of tumorigenesis-related pathways.

## INTRODUCTION

Colorectal cancer (CRC) is a leading cause of cancer-related mortality worldwide, and its incidence has increased rapidly in recent years in China [1, 2]. The etiology of CRC includes genetic factors; external environment factors, such as dietary structure, smoking, drinking and other unhealthy lifestyles; and internal environment factors, characterized by intestinal microbiota disturbance, immunological derangement and the activation of tumor-related signaling pathways. Accumulating evidence links CRC with the intestinal microbiota [3–5].

There are about 100 trillion bacteria in the human intestine, which constitute the intestinal microbiome [6]. This large group of bacteria is termed intestinal symbiotic bacteria, because of their mutualistic and interdependent relationship with the human body during the long period of co-evolution [7]. The intestinal microbiota community is closely related to the development of CRC via their influence on the physiological functions of the colorectum and even the entire digestive system [7, 8].

Our previous research indicated a structural imbalance in the gut microbiota in patients with advanced colorectal adenoma (CRA), which was represented by a reduction of butyrate-producing bacteria and an increase of opportunistic pathogens compared with healthy controls [2]. However, the exact role of the intestinal microbiota during the occurrence and development of CRC requires further exploration. In addition, whether the microbiota imbalance could be restored by certain chemicals or factors remains unknown.

Berberine (BBR) is an isoquinoline alkaloid and a pharmacological component of the Chinese herb *Coptis chinensis*, which has been used to treat intestinal infections, particularly bacterial diarrhea, for thousands of years in China [9]. Recently, BBR has been reported to modulate microbiota structures, which contributed to improving obesity and insulin resistance in mice [10, 11]. However, BBR has a poor oral bioavailability and is difficult to absorb into the bloodstream from the intestines [12, 13], which has contributed to the lack of data concerning the mechanism of BBR's action. Here, we hypothesized that BBR might modulate intestinal microbiota to prevent colorectal carcinogenesis.

The current study was undertaken to evaluate the intestinal microbiota disturbance during the colorectal adenoma-carcinoma sequence, using pyrosequencing of the 16S ribosome RNA (rRNA) genome from fecal samples of healthy controls and patients with CRA or CRC. We also attempted to identify the effect on colonic tumorigenesis of the bacteria *Fusobacterium nucleatum* (*F. nucleatum*) and of BBR on *F. nucleatum*-induced tumorigenesis *in vivo*. These findings may provide a more comprehensive understanding of the role of intestinal microbiota during the occurrence and development of CRC and have important implications for the prevention and treatment of CRC.

## MATERIALS AND METHODS

### Human specimen collection

Fecal samples and left colonic tissues were collected from consecutive patients who had undergone colonoscopy or colorectal carcinoma surgery in the Renji Hospital between 1 January 2012 and 30 July 2012. Patients must have met the following inclusion criteria to enter the study: 1) patients were over 50 years of age, because current US, European and Asian guidelines define the age threshold for endoscopy at 50 y [2]; 2) patients had a normal bowel frequency (minimum of once every 2 day and a maximum of twice per day) [14]; and 3) patients underwent colonoscopies with adequate withdrawal time [15] by well-trained gastroenterologists using standard colonoscopy equipment. The exclusion criteria were the same as our previous study [2]. Patients with CRA or CRC confirmed by both colonoscopy and pathological examination were included in the CRA group or CRC group, respectively. Patients without obvious abnormalities were enrolled in the NC (Negative control) group. We enrolled 52 cases in the NC group, 47 patients in the CRA group and 42 patients in the CRC group. All subjects were asked to provide fresh stool samples, which were immediately stored at −80°C for further analysis. The samples were collected and preserved according to our previous study [2].

All procedures were undertaken in accordance with the Declaration of Helsinki. The Ethics Committees in the Renji Hospital at each participating center approved the study protocol. Informed consent was obtained from all of the subjects. An independent data and safety committee monitored the trial and reviewed the results.

### Bacterial strains and culturing

*Fusobacterium nucleatum subsp. nucleatum* ATCC 25586 [16] and *Escherichia coli* MG1655 ATCC 47076 [17] were purchased from American type culture collection (ATCC). *F. nucleatum* were cultured overnight at 37°C under anaerobic conditions (DG250, Don Whitley Scientific, West Yorkshire, UK) in brain heart infusion (BHI) broth supplemented with hemin, K_2_HPO_4_, vitamin K_1_, and L-Cysteine [18]. The commensal *E. coli* strain MG1655 was used as the non-pathogenic control and was cultured in Luria-Bertani (LB) medium [17]. Bacteria were centrifuged after culturing, and suspended in phosphate buffered solution (PBS) for animal experiments.

### Chemicals

We injected 1,2-dimethylhydrazine (DMH) into mice to model the occurrence of a colonic tumor [19]. DMH is one of the two isomers of dimethylhydrazine. DMH is a potent carcinogen that acts as a DNA methylating agent and it is used to induce colon tumors in experimental animals. DMH and BBR were both obtained from Sigma Chemical Co. (St. Louis, MO, USA) and were prepared by dissolving them in PBS.

### Animal experiments

All mice were maintained in specific pathogen-free (SPF) conditions at the Animal Experimental Center of Tongji University. Fifty male C57BL/6-*APC^Min/+^* mice (The *APC^Min/+^* (adenomatous polyposis coli, APC; multiple intestinal neoplasia, Min) mouse is a popular animal model for studies of human colon cancer) were randomly and equally separated into five groups: Control (Ctr), *F. nucleatum* (Fn), *E. coli* (Ec), BBR and Fn+BBR. Ninety male wild-type C57BL/6 were separated into nine groups: Control (Ctr), DMH, Fn, Fn+DMH, Ec, Ec+DMH, BBR, BBR+DMH and BBR+Fn+DMH. Experiments were performed after adaptive breeding for 1 week. Bacteria were fed at 10^9^ colony forming units (CFU) suspended with 0.1 ml PBS per day. DMH was injected subcutaneous at a dose of 20 mg/kg once weekly. BBR was administrated by gavage at a dose of 100 mg/kg two hours after bacterial feeding. PBS was used as the control treatment. All treatments were performed for 8 weeks to ensure aberrant crypt foci (ACF) formation or 20 weeks to ensure tumor formation. ACF are clusters of abnormal tube-like glands in the lining of the colon and rectum. ACF form before colorectal polyps and are one of the earliest changes seen in the colon that may lead to cancer. Study end points included the occurrence of colonic ACF or tumors, and changes in the lumen microbial structures and the expression of mucosa tumor immune cytokines in mice of different groups. The ACF and colon tumors were identified by methylene blue staining and hematoxylin-eosin staining.

### DNA preparation

The E.Z.N.A. Stool DNA Kit (Omega Bio-Tek, Inc., Norcross, GA, USA) was used to extract DNA from 200 mg of fecal samples for wild-type C57BL/6 mice, C57BL/6-*APC^Min/+^* mice and humans, according to the manufacturer's instructions. The QIAamp DNA Mini Kit (QIAGEN, Hilden, Germany) was used to isolate DNA from colonic tissues, with additional bead-beating steps on a FastPrep-24 (MP Biomedicals, Santa Ana, CA, USA), as previously described [20, 21].

### 454 FLX pyrosequencing

To investigate the microbiota community composition in fecal or colon tissues, V1~V3 hypervariable regions of the 16S rRNA gene were amplified by polymerase chain reaction (PCR) using universal primers (27F 5′- AGAGTTTGATCCTGGCTCAG - 3′, 533R 5′ - TTACCGC GGCTGCTGGCAC - 3′) incorporating the FLX Titanium adaptors and a sample barcode sequence [2, 22]. The prepared DNA was pyrosequenced by using a Roche 454 GS FLX, in accordance with the manufacturer's instructions.

### Taxonomic analysis

The obtained sequences were analyzed using MOTHUR software (version 1.14). The quality control and specific taxonomic procedures were performed similarly to our previous study [2]. According to the taxonomy information, differences among specimens or among clinical groups were analyzed using Metastats and principal component analysis (PCA).

### Real-time quantitative PCR assay

DNA from each specimen was subjected to real-time quantitative PCR (qPCR) assays to determine the amounts of total bacteria and *F. nucleatum* by detecting the 16S genes. The qPCR assay was performed in triplicate with a SYBR Premix Ex Taq (Takara) on an ABI 7900HT Sequence Detection System (Applied Biosystems, Foster City, CA, USA). Amplifications were performed under the following reaction conditions: 10 min at 95°C, followed by 40 cycles of denaturation at 95°C for 15 sec, annealing at the required temperature for 40 sec and at 60°C for 1 min. Cycle threshold (C_T_) values were calculated using the automated settings for SDS 2.2 (Applied Biosystems). The primer sequences of 16S and *F. nucleatum* for each assay were the same as those used in our previous studies [2, 16]. Relative abundance was calculated by the ΔΔC_T_ method.

### Bio-Plex Pro™ mice mucosa cytokine assays

Bio-Plex Pro™ cytokine assays (BIO-RAD, Hercules, CA, USA) are magnetic bead-based multiplex assays designed to measure multiple members of this diverse group of proteins in a minimal volume of matrix, such as serum, plasma, tissue supernatant and other biological fluids [23]. The use of magnetic beads allows researchers to automate the wash steps on a Bio-Plex Pro wash station. Magnetic separation offers greater convenience, productivity and reproducibility compared with vacuum filtration.

We used mice colonic tissue lysates extracted using RIPA with PMSF (1:100), Protease Inhibitor Cocktail (1:100) and Phosphatase Inhibitor Cocktail (1:100). The Bio-Plex Pro™ suspension array system is operated around three core elements of xMAP technology fluorescently dyed microspheres to permit discrimination of individual tests within a multiplex suspension; a dedicated flow-cytometer with two lasers and associated optics to measure the different molecules bound to the surface of the beads; and a high-speed digital signal processor to manage the fluorescence data efficiently.

### Western blot analysis

Proteins were extracted from mice colonic tissue and quantified using a Pierce™ BCA Protein Assay Kit (Thermo Scientific™). Proteins were subjected to 10% SDS-PAGE and transferred to polyvinylidene fluoride (PVDF) membranes, and blocked with 5% bovine serum Albumin in PBS containing 0.1% Tween-20. Equal protein loading was controlled by Ponceau Red staining of the membranes. Blots were probed using primary antibodies. A GAPDH antibody was used as a control for whole-cell lysates. Antibodies were purchased from Cell Signaling Technology Inc., except for GAPDH (Kangchen), and AKT2 and p-AKT2 (Abcam). After washes, membranes were incubated with the appropriate HRP-conjugated secondary antibodies (Kangchen), and blots were detected using the Chemiluminescent Western Blot Detection (Thermo Scientific™).

### Statistical analysis

The Mann-Whitney *U* and One-Way ANOVA tests were performed using SPSS 17.0 software (SPSS Inc., Chicago, IL, USA) and MOTHUR, when comparing the intestinal microbiota structures among the different human subject groups or mice groups with various treatments. Student's *t*-test was used to describe colon ACF or tumors, and the expression of mucosa tumor-immune cytokines, compared with corresponding mice groups, according to the study design. Two-sided *P*-values <0.05 were considered statistically significant.

## RESULTS

### Structural transformation of lumen microbiota during the colorectal adenoma-carcinoma sequence

We first analyzed the fecal microbial community among negative control subjects, CRA and CRC patients. A total of 2,296,326 optimized sequences were obtained with an average of 437 base pairs per sequence. The sequences were clustered using 3% dissimilarity as an indicator of an operational taxonomic unit (OTU). There were no significant differences among the relative microbiota abundance of the fecal samples from negative subjects group (FN, *n* = 52), CRA group (FA, *n* = 47) and CRC group (FC, *n* = 42) when comparing the richness estimators (Ace and Chao) and the diversity estimators (Shannon and Simpson). The rarefaction curve showed the ends of the curve per sample tending towards stability (Supplementary Figure 1), which indicated that the pyrosequencing data generated in this study were sufficient to reflect the diversity of the intestinal microbiota.

**Figure 1:**
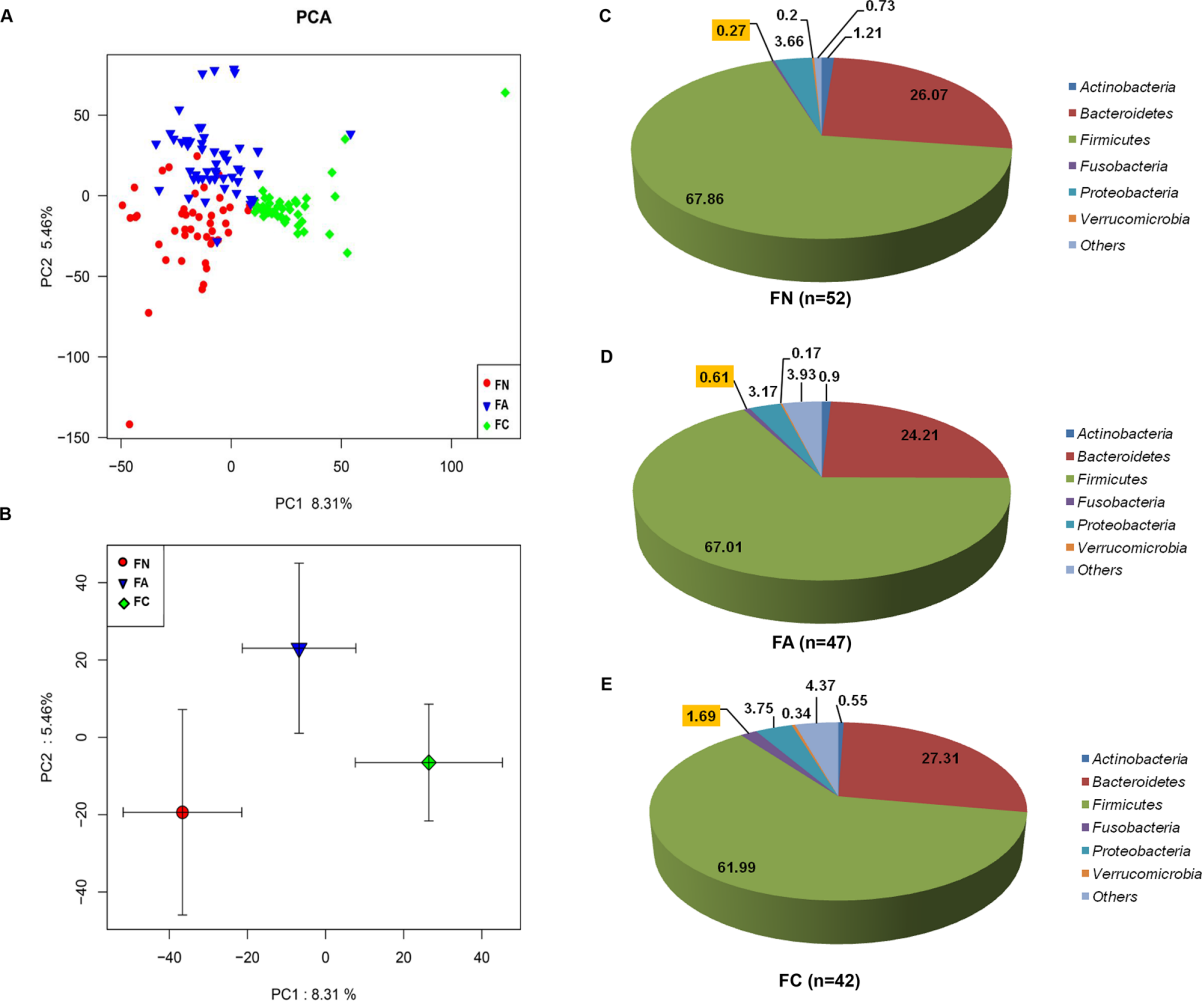
Intestinal microbiota structures among the FN group (*n* = 52), FA group (*n* = 47) and FC group (*n* = 42) **A.** Principal component analysis (PCA) scores plot based on unweighted UniFrac metrics. Each symbol represents a sample. **B.** PCA scores plot based on unweighted UniFrac metrics. Each point represents the mean principal component scores of all populations in a different group, and the error bar represents the standard deviation. **C.** Relative abundance of lumen microbiota distribution in the negative control (FN) group (*n* = 52) at the phylum level. **D.** Relative abundance of lumen microbiota distribution in the CRA (FA) group (*n* = 47) at the phylum level. **E.** Relative abundance of lumen microbiota distribution in the CRC (FC) group (*n* = 42) at the phylum level.

Principal component analysis (PCA), which is based on unweighted Unifrac metrics, showed alteration of fecal intestinal microbiota communities in patients with CRA or CRC compared with the negative controls (Figure 1A and 1B). The principal components of fecal gut microbiota were significantly different in the three groups. The FA group was located between the FN and FC both on the X-axis in the PC1 direction and on the Y-axis in the PC2 direction (Figure 1A and 1B), which indicated that structural transformation of fecal gut microbiota might occur from normal colorectal tissues to CRA and then to CRC (*P* < 0.05, ANOVA).

More than half of the microbial community comprised the *Firmicutes* phylum: 67.86% in the FN group (*n* = 52), 67.01% in the FA group (*n* = 47), and 61.99% in the FC group (*n* = 42). The next most abundant phyla were *Bacteroidetes*, *Proteobacteria*, *Fusobacteria*, *Actinobacteria*, and *Verrucomicrobia*. These six bacterial phyla accounted for more than 90% of the microbial community in the three groups. Furthermore, the proportions of the *Fusobacterial* phylum showed a significant, gradually increase during the colorectal adenoma-carcinoma sequence: from normal colorectal tissue (0.27% in the FN group) to adenoma tissues (0.61% in the FA group) and to CRC tissues (1.69% in the FC group) (*P* = 0.016; 2.26 fold change (FA/FN) and 6.26 fold change (FC/FN), respectively, Figure 1C–1E).

We analyzed the fecal microbial community among the three groups at the genera level. A heatmap was generated with 55 genera whose relative abundance was more than 0.1% of the total bacteria in the stool samples (Supplementary Figure 2A). The data revealed that the levels of opportunistic pathogens, such as *Fusobacterium*, *Streptococcus*, and *Enterococcus* spp. increased during the colorectal adenoma-carcinoma sequence in the lumen (all *P* < 0.05, Table 1 and Supplementary Figure 2B).

**Table 1:** Significant differences in some genera identified among the fecal microbiota of the FN group (*n* = 52), FA group (*n* = 47) and FC group (*n* = 42)

*Phylum*	*Genus*	FN (*n* = 52)	FA (*n* = 47)	FC (*n* = 42)	*P* value	Tendency from FN-FA-FC
*Actinobacteria*	*Actinomyces*	0.09 ± 0.02	0.07 ± 0.02	0.04 ± 0.02	<0.001	↓
*Actinobacteria*	*Bifidobacterium*	0.05 ± 0.01	0.03 ± 0.01	0.01 ± 0.00	0.008	↓
*Firmicutes*	*Blautia*	14.18 ± 1.94	12.28 ± 2.02	3.01 ± 0.55	<0.001	↓
*Firmicutes*	*Clostridium*	2.08 ± 0.41	0.88 ± 0.20	0.31 ± 0.09	<0.001	↓
*Firmicutes*	*Dorea*	1.70 ± 0.27	1.45 ± 0.35	0.29 ± 0.06	<0.001	↓
*Firmicutes*	*Lactobacillus*	1.79 ± 1.16	0.70 ± 0.37	0.14 ± 0.09	0.011	↓
*Firmicutes*	*Roseburia*	2.67 ± 0.50	1.22 ± 0.27	0.75 ± 0.15	0.003	↓
*Firmicutes*	*Eubacterium*	0.08 ± 0.03	0.04 ± 0.02	0.02 ± 0.01	0.013	↓
*Fusobacteria*	*Fusobacterium*	0.26 ± 0.18	0.61 ± 0.29	1.22 ± 0.50	0.031	↑
*Proreobacteria*	*Escherichia-Shigella*	1.32 ± 0.32	1.82 ± 0.41	2.74 ± 0.66	0.025	↑
*Firmicutes*	*Coprococcus*	0.44 ± 0.07	0.73 ± 0.12	1.24 ± 0.27	0.034	↑
*Firmicutes*	*Streptococcus*	2.33 ± 0.50	3.75 ± 0.65	5.55 ± 1.22	0.016	↑
*Firmicutes*	*Enterococcus*	0.26 ± 0.15	1.42 ± 0.51	2.75 ± 0.92	0.004	↑

Note: 1. Relative contribution of a genus was calculated as percentage of the sequences of this genus to all sequences in this population. 2. Mean ± SEM (standard error of mean) was calculated according to percentage of the sequences of this genus to all sequences in each individual. 3. The differences of relative abundances were calculated using ANOVA assay. Two-sided *P*-values < 0.05 were considered statistically significant. The increasing or decreasing tendency during FN-FA-FC sequence was represented as ↓ or ↓, respectively.

### Structural transformation of colonic mucosa microbiota during the colorectal adenoma-carcinoma sequence

The conclusions from our fecal pyrosequencing data agreed with previous reports [2, 3, 24]. However, there were few reports concerning the mucosa microbial structures transformed during the colorectal adenoma-carcinoma sequence [25, 26]. To identify the changed structures in the mucosa microbiota, pyrosequencing was also performed with DNA samples extracted from 98 colonic tissues, including 37 cases with normal colonic mucosa from negative control subjects (MN), 30 cases of adenoma tissues (MA), and 31 cases of carcinoma tissues (MC). From the pyrosequencing analysis, similar trends in the variation of mucosal microbiota were observed to those in the lumenal microbiota (Supplementary Figure 2B and 2C).

### Enrichment of *Fusobacterium nucleatum* during the colorectal adenoma-carcinoma sequence

To further clarify the enrichment of *F. nucleatum* during the adenoma-carcinoma sequence pathway, we used a targeted qPCR assay to determine its abundance in feces or colonic tissues from various populations. QPCR measured the abundance of *Fusobacterium*, and the data was consistent with the results from the pyrosequencing data (Spearman correlation *r* = 0.827, *P* < 0.05). The qPCR data showed that *Fusobacterium nucleatum* gradually increased with the sequence from healthy controls to CRA and eventually to CRC, both in fecal and mucosal samples (Supplementary Figure 3A and 3B). Our results demonstrated that *F. nucleatum* accumulated in the human intestine at the early stage of colon cancer development, and might play a role during the colorectal adenoma-carcinoma sequence.

### *Fusobacterium nucleatum* promoted colon tumorigenesis in mice

**Figure 2:**
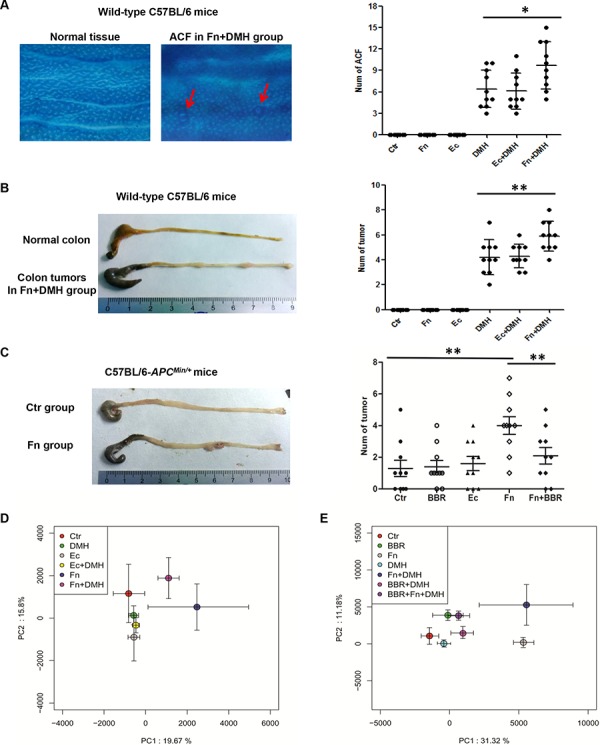
Intestine tumorigenesis and lumenal microbiota structures in mice with treated with *Fusobacterium nucleatum* or BBR **A.** Colon ACF in wild-type C57BL/6 mice observed by staining with 0.2% methylene blue under a light microscope at 8 weeks (*n* = 10 per group). ACF are highlighted with red arrows. **B.** Intestine tumorigenesis in wild-type C57BL/6 mice at 20 weeks (*n* = 10 per group). **C.** Intestine tumorigenesis in C57BL/6-*APC^Min/+^* mice at 8 weeks (*n* = 10 per group). Two-sided *P*-values < 0.05 were considered statistically significant. *P < 0.05 **P < 0.01. **D.** *F. nucleatum* colonization altered intestinal microbiota structures in mice. **E.** BBR modulated the microbiota disturbance induced by *F. nucleatum.* Each point represents the mean principal component scores of all population in the different groups, and the error bar represents the standard deviation.

**Figure 3:**
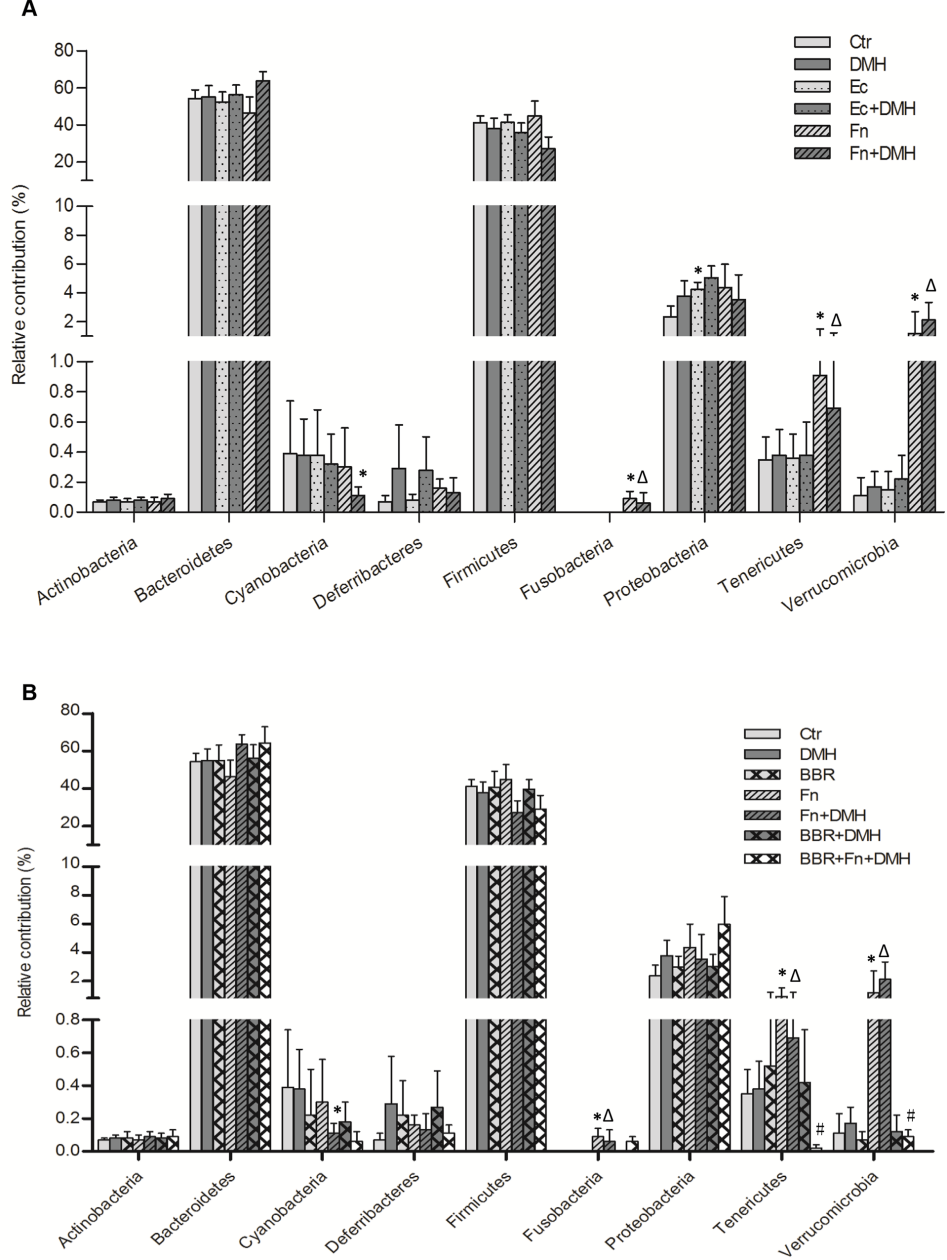
Lumen microbiota distribution at the phylum level in mice treated with A. bacteria or B. BBR The relative contribution of a phylum was calculated as the percentage of the sequences of this phylum to all sequences in this population. Mean ± SEM (standard error of mean) was calculated according to percentage of the sequences of this phylum to all sequences in each individual. The differences in relative abundances were calculated using Mann-Whitney U test. Two-sided *P*-values < 0.05 were considered statistically significant. *P < 0.05, compared with Ctr group; Δ*P* < 0.05, compared with DMH group; #*P* < 0.05, compared with Fn+DMH group.

Colon tumorigenesis was analyzed by calculating the number of ACF and colon tumors in wild-type C57BL/6 mice and C57BL/6-*APC^Min/+^* mice. The mice with DMH injected all generated obvious ACF in their colons (Figure 3A), and mice administered with both DMH and *F. nucleatum* (Fn+DMH) generated the largest numbers of ACF (Figure 2A) and colon tumors (Figure 2B). Interestingly, all the *APC^Min/+^* mice administered with *F. nucleatum* for 8 weeks developed more colon tumors than the *APC^Min/+^* control (Ctr) and the *Escherichia coli* (Ec) bacteria control mice (*P* < 0.01, Figure 2C). These data suggested that *F. nucleatum* might accelerate tumorigenesis *in vivo*.

### *Fusobacterium nucleatum* altered lumen microbial structures in mice

We next pyrosequenced the lumenal microbial components in the different treatment groups of wild-type C57BL/6 mice. PCA of lumenal microbiota was performed among six groups of mice. As shown in Figure 2D, the microbial structures of the lumen in the mice after *F. nucleatum* treatment were significantly different from mice without bacterial administration or those receiving *E. coli* administration (*P* < 0.05, ANOVA), indicating that in addition to promoting colon tumorigenesis, introduction of *F. nucleatum* altered the lumenal microbial structures in mice.

A total of 952 OTUs were found in the wild-type C57BL/6 mice. Almost all of the OTUs were represented by the following phyla: *Bacteroidetes*, *Firmicutes*, *Proteobacteria*, *Verrucomicrobia*, *Cyanobacteria*, *Tenericutes*, and Unclassified bacteria, which were similar to humans. There was no *Fusobacteria* colonization in the lumen of wild-type C57BL/6 mice. However, in addition to promoting colon tumorigenesis, colonization by *F. nucleatum* dramatically altered the lumen microbial structures by increasing the *Tenericutes* and *Verrucomicrobia* in the mice (Figure 3A).

### BBR could reverse the *F. nucleatum*-induced lumenal microbiota imbalance and colon tumorigenesis in mice

**Figure 4:**
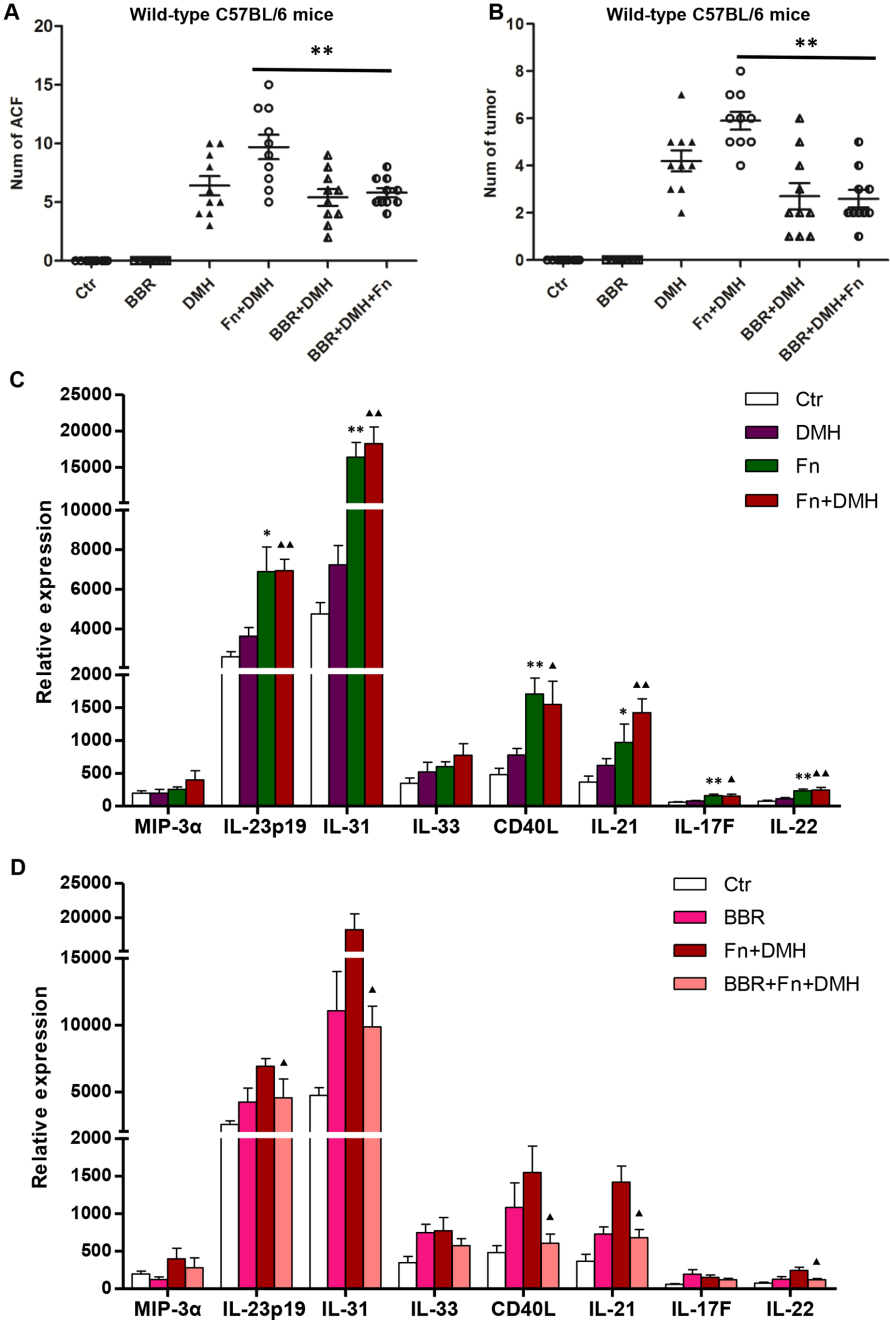
BBR inhibited colon tumorigenesis induced by *F. nucleatum* in mice by modulating the expression of mucosa tumor-immune cytokines **A.** Colon ACF and **B.** tumors counted in wild-type C57BL/6 mice with BBR intervention at different times. Two-sided *P*-values < 0.05 were considered statistically significant. **P < 0.01. **C.** *F. nucleatum* colonization altered the expression of mucosa tumor-immune cytokines in mice. *P < 0.05 and **P < 0.01, compared with the Ctr group; ▲*P* < 0.05 and ▲▲*P* < 0.01, compared with the DMH group. **D.** BBR modulated the mucosa tumor-immune cytokines secretion induced by *F. nucleatum*. ▲*P* < 0.05 and ▲▲*P* < 0.01, compared with the Fn+DMH group.

Previous studies have reported that some chemicals, such as dietary fiber [2, 27], folate [28, 29] or BBR [30–32] had a preventative effect on colonic tumorigenesis. Recently, BBR has been reported to improve obesity and insulin resistance in mice by changing microbiota structures [33]. Therefore, we next introduced BBR into mice to identify whether it could rescue the *F. nucleatum*-induced microbiota disturbance. The microbial structures alteration, which was characterized by the increase in *Tenericutes* and *Verrucomicrobia*, was dramatically reversed in *F. nucleatum* infected mice after BBR intervention (Figure 2E and Figure 3B). These findings suggested that BBR intervention significantly blocked the *Fusobacteria*-induced lumen microbiota imbalance. In addition, C57BL/6-*APC^Min/+^* mice treated with BBR developed significantly fewer colon tumors compared with mice fed without BBR after *F. nucleatum* treatment (Figure 2C). The same phenomena were observed in wild-type C57BL/6 mice (Figure 4A and 4B).

### BBR might modulate the *F. nucleatum*-mediated increase of tumor-immune cytokine secretion in mice

It has been reported that *F. nucleatum* potentiates intestinal tumorigenesis by modulating the tumor-immune microenvironment in mouse models [24]. In this study, we detected changes in the mucosal cytokines by performing Bio-Plex Pro™ Assays to explore the colon microenvironment alteration in mice after *F. nucleatum* or BBR treatment. As shown in Figure 4C, the levels of IL-17F/21/22/23/31 and CD40L increased after the introduction of *F. nucleatum* in mice, indicating that colonization by *F. nucleatum* stimulated the secretion of immune cytokines. Furthermore, the increased secretion of IL-21/22/31 and CD40L were reversed after the addition of BBR in *F. nucleatum*-treated mice (Figure 4D). The data indicated that BBR might modulate the increased secretion of mucosa immune cytokines in *F. nucleatum* colonized mice.

### BBR could block *F. nucleatum*-induced colon tumorigenesis in mice by inhibiting the activation of JAK/STAT and MAPK pathways

**Figure 5:**
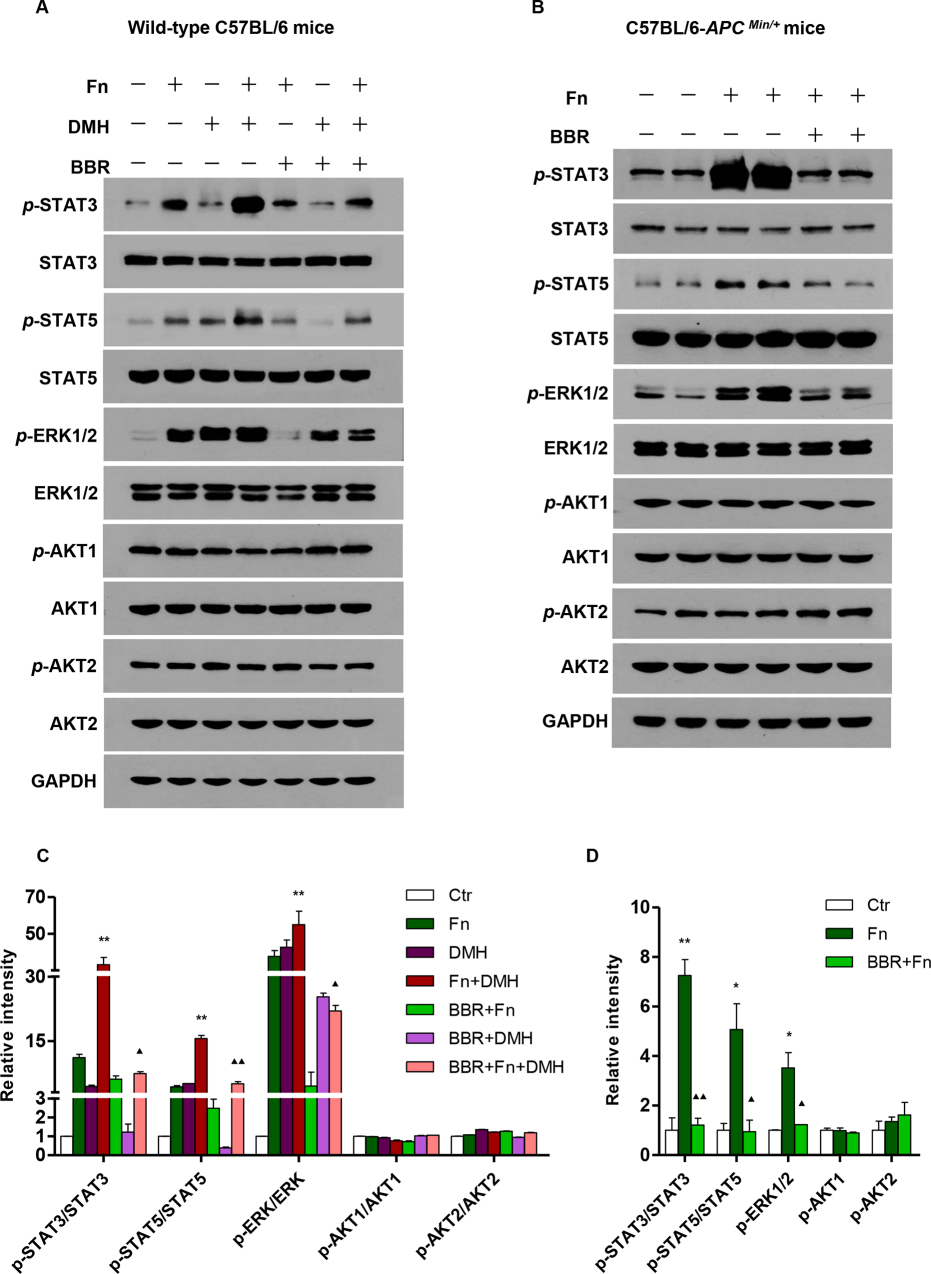
BBR inhibited colon tumorigenesis induced by *F. nucleatum* in mice by modulating JAK/STAT and MAPK pathways Western blot assays were performed to measure the expressions of STAT3, STAT5, ERK1/2, AKT1/2, p-STAT3, p-STAT5, p-ERK1/2 and p-AKT1/2 with different treatments in wild-type C57BL/6 mice **A.** and in C57BL/6-*APC^Min/+^* mice **B, C.** The summarized analysis of the western blot is shown in wild-type C57BL/6 mice with different treatments. *P < 0.05 and **P < 0.01, compared with the Ctr group; ▲*P* < 0.05 and ▲▲*P* < 0.01, compared with the Fn+DMH group. **D.** The summarized analysis of western blot is shown in C57BL/6-*APC^Min/+^* mice with different treatments. *P < 0.05 and **P < 0.01, compared with the Ctr group; ▲*P* < 0.05 and ▲▲*P* < 0.01, compared with the Fn group.

Numerous studies have indicated some tumor-immune cytokines play crucial roles in tumor development and progression by activating specific signaling pathways [34–36]. It has been reported that IL-21/22/31 and CD40L activate the JAK/STAT, MAPK/ERK and PI3K/AKT pathways [25, 26]. Therefore, we next examined whether *F. nucleatum*-induced increases in the secretion of these cytokines via activation of JAK/STAT, MAPK/ERK and PI3K/AKT pathways. As shown in Figure 5, the expressions of *p*-STAT3, *p*-STAT5 and *p*-ERK1/2 increased after introduction of *F. nucleatum* both in Wild-type C57BL/6 mice and C57BL/6-*APC^Min/+^* mice, indicating that *F. nucleatum* colonization may activate JAK/STAT and MAPK/ERK pathways. Furthermore, the *F. nucleatum*-induced increases in *p*-STAT3, *p*-STAT5 and *p*-ERK1/2 expressions were significantly reduced by BBR treatment in mice. The data indicated that BBR treatment significantly blocked the *Fusobacteria*-induced activation of the JAK/STAT and MAPK/ERK pathways.

## DISCUSSION

Numerous studies have shown that the intestinal microbial structures are different in patients with CRC compared with healthy subjects [3, 18, 37]. Our results demonstrated that the intestinal microbial structures were altered in the lumen and the mucosa during the progression of the colorectal adenoma-carcinoma sequence. These alterations were characterized by reductions in some beneficial bacteria, such as *Actinomyces*, *Bifidobacterium*, *Lactobacillus*, butyrate-producing bacteria (including *Clostridium*, *Rosuburia*, *Eubacterium*, *Blautia*, and *Dorea* spp.), and by enrichment of opportunistic pathogens, such as *Fusobacterium*, *Escherichia-Shigella*, *Coprococcus*, *Streptococcus* and *Enterococcus* spp. The data indicated that these bacteria might mediate the progression of CRC.

In line with other two research teams from America and Canada who reported the enrichment of *Fusobacterium* spp. in CRC tissue [16, 38, 39], our clinical data showed that *Fusobacteria* and *Fusobacterium* spp. accumulated in the human intestine during the colorectal adenoma-carcinoma sequence. More importantly, our data revealed that *Fusobacterium* expression in the mucosa was consistent with that in feces; therefore, fecal samples may replace tissue specimens as a simpler and more practical diagnostic method for the early detection of *Fusobacterium* enrichment [40].

In this study, we clarified that colonization of *F. nucleatum* in the intestine promoted the onset of colonic tumors *in vivo*. Kostic reported that *F. nucleatum* potentiated intestinal tumorigenesis by modulating the tumor-immune microenvironment [24]. However, the mechanism of *F. nucleatum*-mediated colorectal tumorigenesis is still poorly understood. We hypothesized that *F. nucleatum* might promote colorectal tumorigenesis because 1) *F. nucleatum* disturbs the intestinal microbiota balance via an increase in opportunistic pathogens and decreased probiotics; and 2) *F. nucleatum* induces tumor-related immune cytokine secretion, such as IL-21/22/31 and CD40L, and activates the JAK/STAT and MAPK/ERK pathways, which have been reported to promote tumorigenesis. Both IL-21 [41] and CD40L [42] have been linked to colorectal tumorigenesis. IL-22 may play a prominent role in the progression of CRC by activation of STAT3 (Signal Transducer and Activator of Transcription 3) [43]. IL-31 modulates cell proliferation and migration, suggesting a role in the regulation of the intestinal barrier function [36]. Both STAT3 and STAT5 increase tumor cell proliferation, survival and invasion, while suppressing anti-tumor immunity [44]. Activated MAPK/ERK plays an important part in progression of colorectal cancer [45].

The lumenal microbial community, combined with the mucosal immune system, determines the intestinal homeostasis balance, which represents the intestinal environment to some extent. There is growing evidence that the balance between symbiotic bacteria and the host defense response from the mucosa plays an essential role in the genesis, growth and metastasis of CRC [46–48]. Immune dysfunction might be an important mediating factor for the effects of intestinal symbiotic bacteria on inflammation and tumorigenesis. When intestinal microbial structures shift because of exterior factors, the link with the immune system could be broken, resulting in immune dysfunction, which ultimately leads to the occurrence and development of CRC [49, 50].

Some factors such as dietary fiber [2, 27], folate [28, 29] or BBR [30–32] prevent colonic tumorigenesis. BBR decreased the relative contribution of *Firmicutes* and *Bacteroidetes* in mice fed with a high-fat diet, and upregulated the expression of fasting-induced adipose factor genes in both intestinal and adipose tissues, which indicated that the antimicrobial activity of BBR contributed to its anti-obesity effects [12, 33]. In this study, we found that BBR not only reversed the microbiota imbalance induced by *F. nucleatum*, but also blocked the secretion of mucosal immune cytokines and the activation of the JAK/STAT and MAPK/ERK pathway, which was stimulated by *F. nucleatum in vivo*. Our data revealed a role for BBR in the prevention of colorectal carcinogenesis.

Recently, it has been reported that microbiota may promote carcinogenesis by activation of TLRs, NF-κB and STAT3 [51–53]. Han et al. demonstrated that *F. nucleatum* promotes colorectal carcinogenesis by modulating the Wnt pathway [54]. In addition, Chinese scientists later reported that BBR attenuates colorectal tumorigensis via inhibition of the Wnt pathway *in vivo* and *in vitro* [30, 31]. In the current study, we determined one of the mechanisms of *F. nucleatum*-mediated colorectal carcinogenesis. Firstly, we found that *F. nucleatum* treatment significantly increased the levels of *p*-STAT3, *p*-STAT5 and *p*-ERK1/2, both in wild-type C57BL/6 mice and C57BL/6-*APC^Min/+^* mice. Furthermore, BBR may attenuate *F. nucleatum*-induced carcinogenesis by blocking the activation of JAK/STAT and MAPK/ERK pathways in mice. However, the specific molecule(s) that participates in the Fusobacterium-induced CRC carcinogenesis, and the mechanism of BBR-mediated blocking of *Fusobacterium*-induced CRC carcinogenesis, should be explored in the future.

In conclusion, *F. nucleatum* might play an important role in the progression of CRC, especially in the colorectal adenoma-carcinoma sequence, where it disturbs the intestinal microbiota balance, alters tumor-related immune cytokine secretion and activates the JAK/STAT and MAPK/ERK pathways. Furthermore, BBR blocks *F. nucleatum*-mediated colorectal tumorigenesis *in vivo* by modulating the microbial balance, and regulating the mucosal immune system, the JAK/STAT pathway and the MAPK/ERK pathway. Further research is needed to reveal which specific cell signals are regulated by *F. nucleatum* at the molecular level.

## SUPPLEMENTARY FIGURES


