# Supplementary material for: Berberine may rescue Fusobacterium nucleatum-induced colorectal tumorigenesis by modulating the tumor microenvironment
Source: Oncotarget. 2015 Sep 12;6(31):32013–26. doi: 10.18632/oncotarget.5166 (PMC4741656; doi:10.18632/oncotarget.5166)
Supplement: Supplementary file 1 [file oncotarget-06-32013-s001.pdf]

SUPPLEMENTARY FIGURES

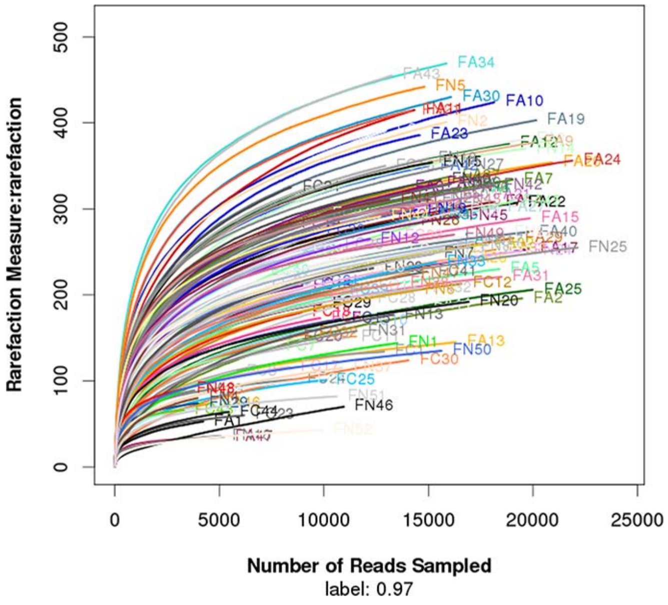

Supplementary Figure S1: The rarefaction curve of FN group (*n* = 52), FA group (*n* = 47) and FC group (*n* = 42).

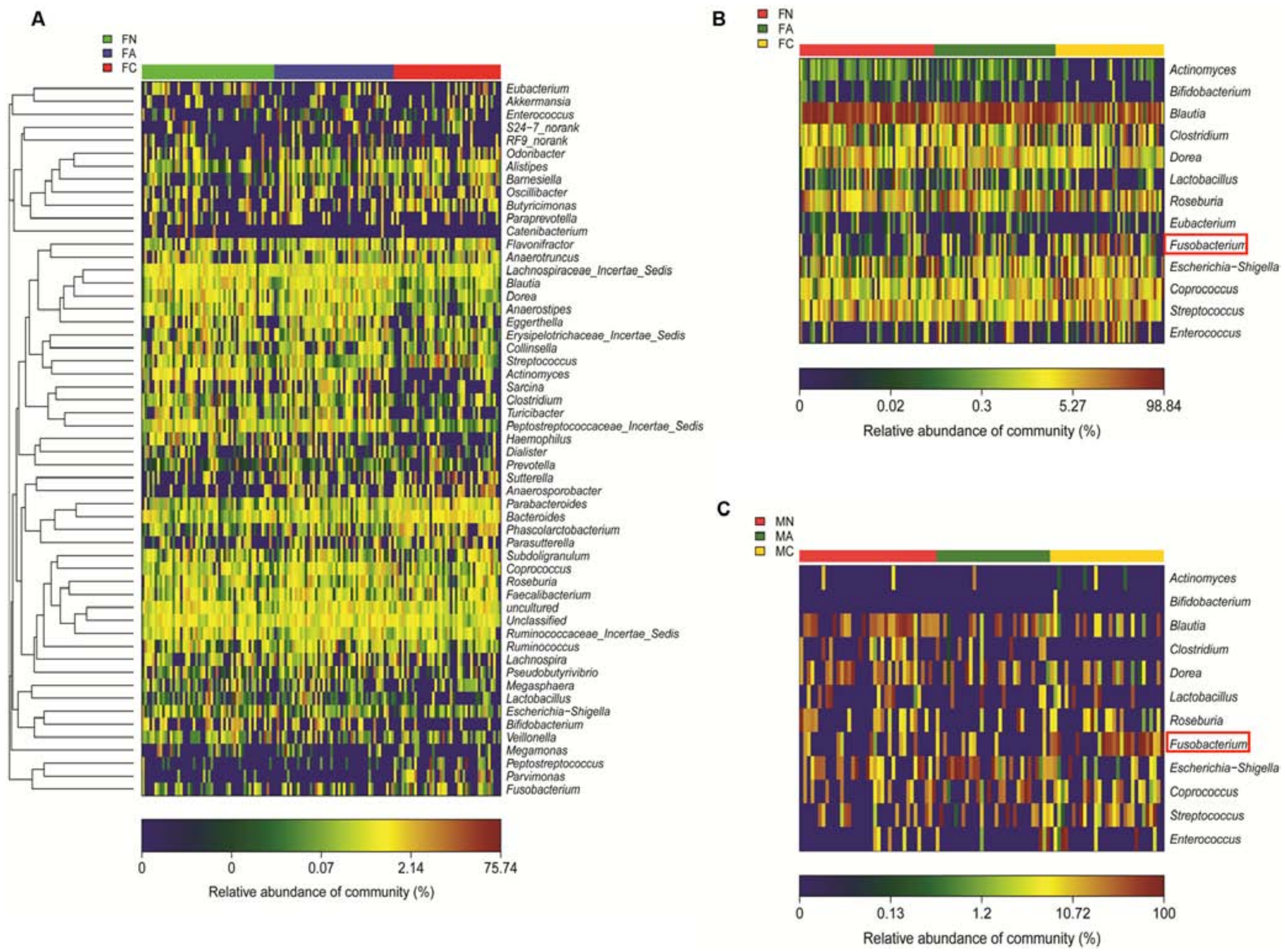

Supplementary Figure S2: The Heatmap of gut microbiota distribution in the genera level. A. The Heatmap of gut microbiota distribution in lumen microbiota in FN (n = 52), FA (n = 47) and FC (n = 42) groups. **B.** Significant differences of 15 genera identified in lumen microbiota in FN (n = 52), FA (n = 47) and FC (n = 42) groups. **C.** Significant differences of 15 genera identified in mucosa microbiota in MN (n = 37), MA (n = 30) and MC (n = 31) groups.

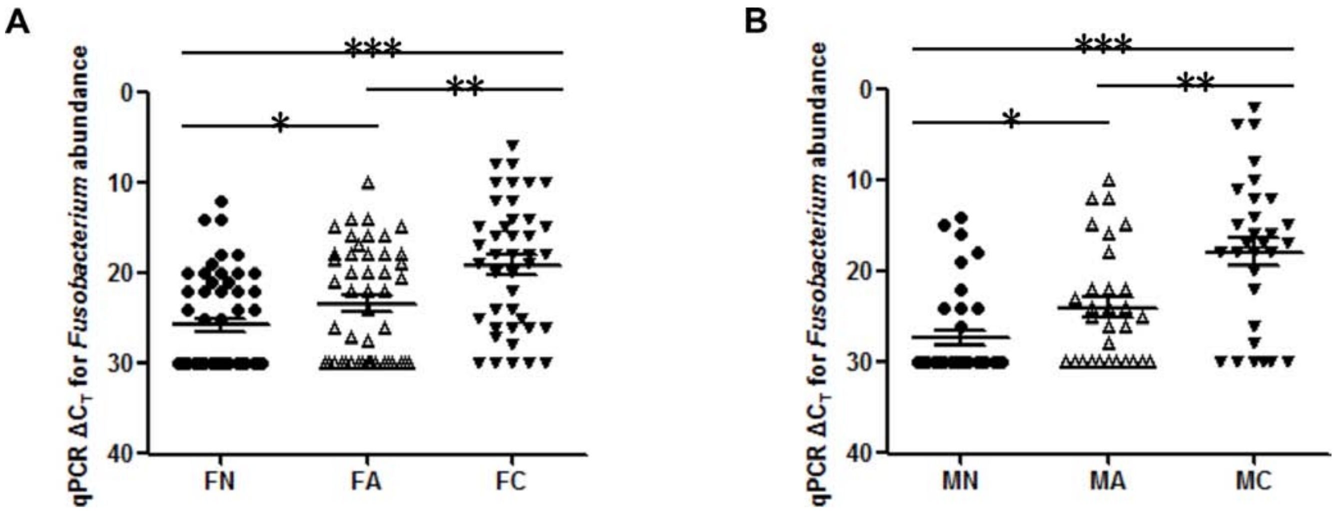

Supplementary Figure S3: Relative abundance of *Fusobacterium nucleatum* in feces or colonic tissues from control subjects or subjects suffered with CRA or CRC. The data shown are mean C_T values from three independent experiments. **A.** Relative abundance of *Fusobacterium nucleatum* in feces from FN ($n = 52$), FA ($n = 47$) and FC ($n = 42$). **B.** Relative abundance of *Fusobacterium nucleatum* in colonic tissues from MN ($n = 37$), MA ($n = 30$) and MC ($n = 31$). Two-sided P-values 0.05 were considered statistically significant. * means $P < 0.05$; ** means $P < 0.01$; *** means $P < 0.001$.
